# Supplementary material for: KSRP specifies monocytic and granulocytic differentiation through regulating miR-129 biogenesis and RUNX1 expression
Source: Nat Commun. 2017 Nov 10;8:1428. doi: 10.1038/s41467-017-01425-3 (PMC5681548; doi:10.1038/s41467-017-01425-3)
Supplement: Supplementary file 1 — Supplementary Information [file 41467_2017_1425_MOESM1_ESM.pdf]

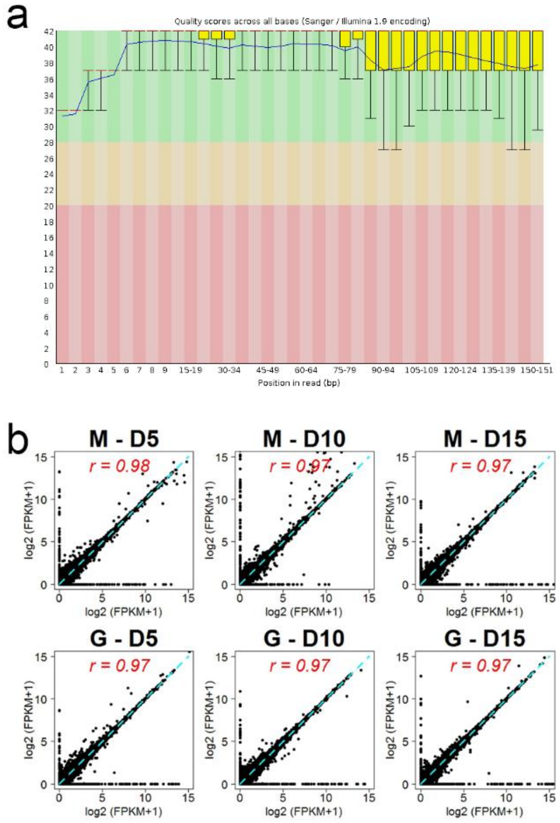

Supplementary Figure 1. Quality control of strand-specific RNA-seq performed in HPCs that differentiated into monocytes or granulocytes.

(a) PHRED quality scores across all bases of the reads. **(b)** Correlation plot of the log2 signals between the duplicate RNA-seq assays. Pearson correlation coefficient (r) were marked in red.

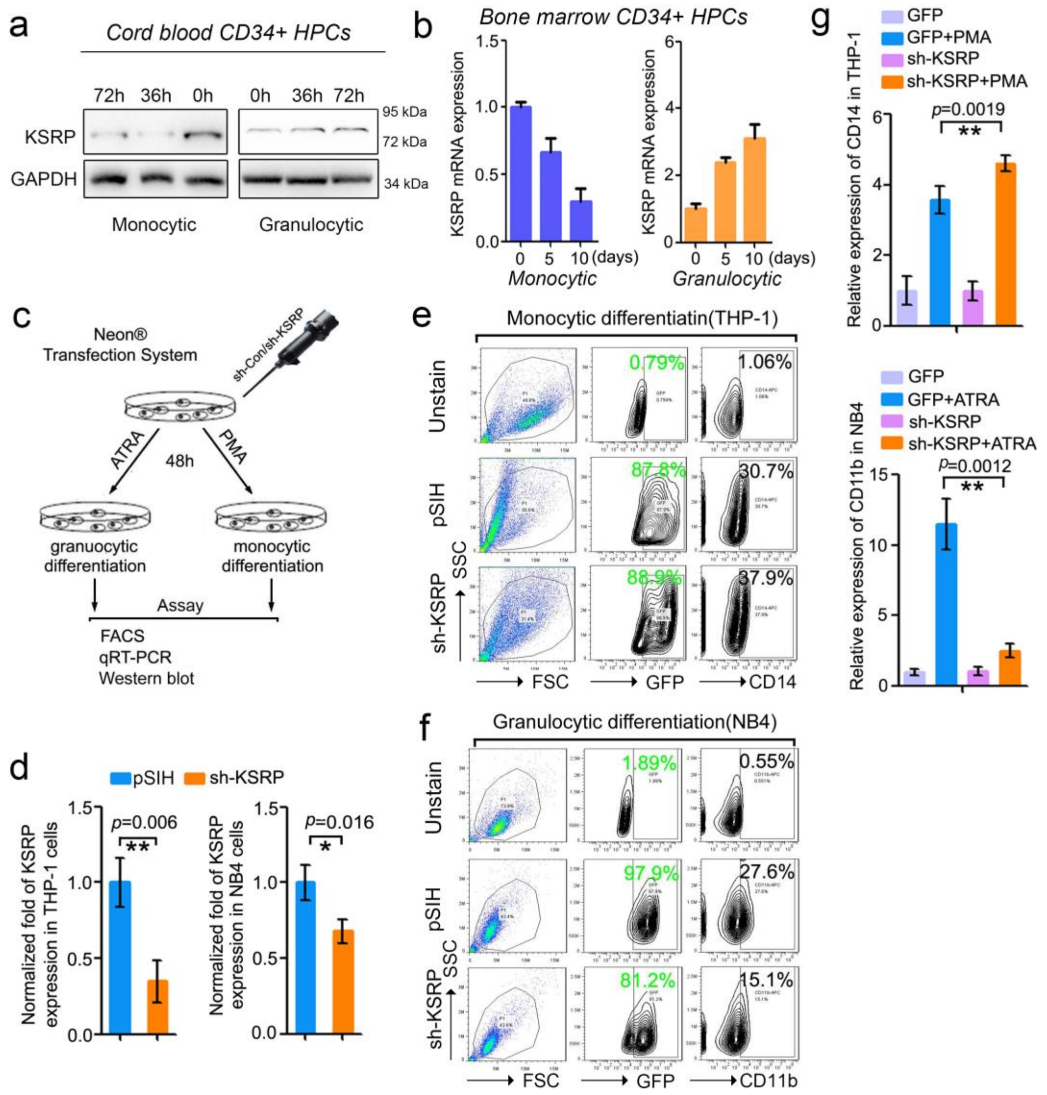

Supplementary Figure 2. KSRP exhibits different functions in cell lines undergoing monocytic or granulocytic differentiation.

(a) Immunoblot analysis of the expression of the KSRP protein in CD34^{+} HPC models of monocyte and granulocyte differentiation from cord blood. **(b)** qPCR analysis of KSRP mRNA expression in monocytic or granulocytic differentiated bone marrow CD34^{+} HPCs. **(c)** Schematic representation of the Neon® transfection system used to knockdown KSRP in THP-1 or NB4 cells and the analysis of KSRP function by FACS, qRT-PCR and immunoblotting. **(d)** qRT-PCR analysis of the KSRP knockdown efficiency. Three technical replicates from a single experiment. **(e)** FACS analysis of the CD14^{+} population in THP-1 cells undergoing monocytic differentiation after Neon® transfection with sh-KSRP. **(f)** FACS analysis of the CD11b^{+} population in NB4 cells

undergoing granulocytic differentiation after Neon® transfection with sh-KSRP. **(g)** qRT-PCR analysis of *cd14* expression in THP-1 cells and *cd11b* expression in NB-4 cells undergoing monocytic or granulocytic differentiation after transduction with lenti-sh-KSRP. Lenti-GFP served as the control. Three technical replicates from a single experiment. Data are shown as means ± s.d.. **P*<0.05, ***P*<0.01, ****P*<0.001, Student's *t*-test.

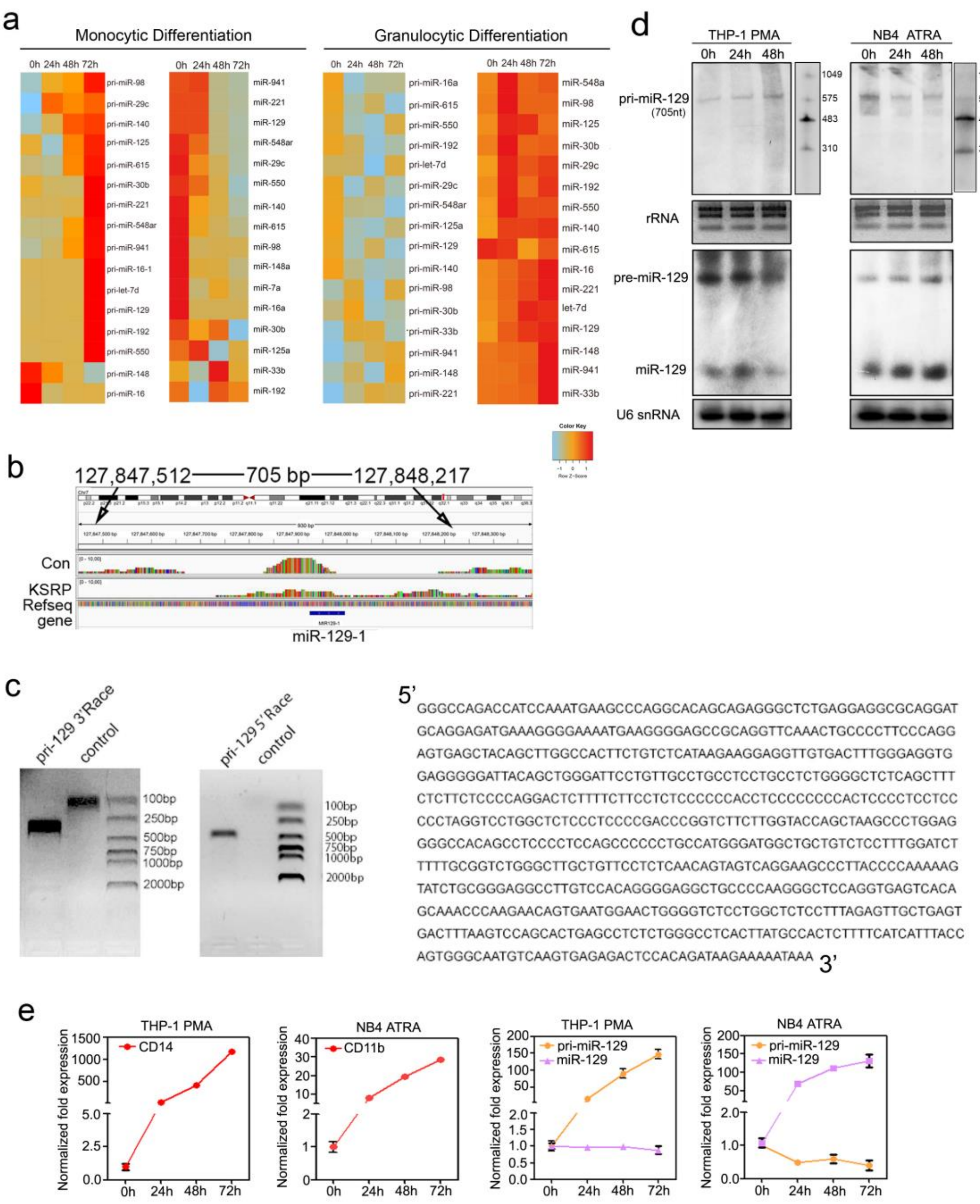

Supplementary Figure 3. KSRP regulates miRNA processing in myeloid cells.

(a) Heat map representation of qRT-PCR data for primary and mature transcripts corresponding to individual miRNAs expressed in THP-1 monocyte cultures or NB4 granulocyte cultures. **(b)** Visualization of RNA-seq coverage across the 705 bp miR-129-1 locus. Coverage (Wiggle) files generated from SAMTOOLS and the annotation file were loaded onto the Integrated Genome Viewer (IGV, developed at the Broad Institute). No reads were mapped to the miR-129-2 locus. **(c)** Left panel: agarose gel electrophoresis showing the results of the 5'-RACE and 3'-RACE assays.

Right panel: the full-length pri-miR-129-1 transcript obtained from the RACE assays. **(d)** Northern blot analysis of miR-129 transcripts in THP-1 cells undergoing monocytic differentiation and NB4 cells undergoing granulocytic differentiation. The upper panel shows the hybridization of pri-miR-129 with DIG-labeled RNA probes; rRNA was used as a loading control. The lower panel shows the hybridization of precursor-miR-129 and mature-miR-129 with isotope-labeled DNA probes; U6 snRNA was used as a loading control. **(e)** qRT-PCR analysis of monocyte-granulocyte markers and primary and mature miR-129 expression in THP-1 monocyte cultures or NB4 granulocyte cultures. Three technical replicates from a single experiment.

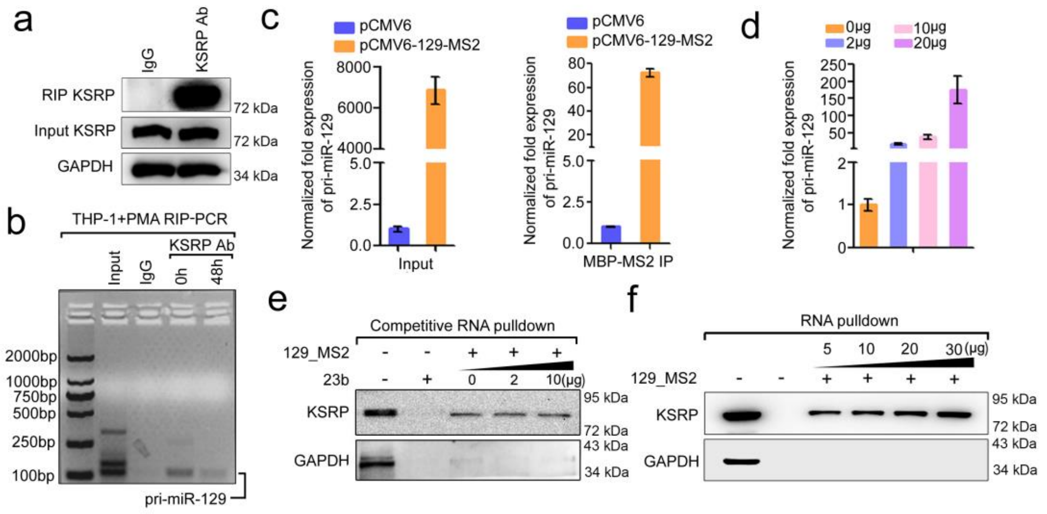

Supplementary Figure 4. KSRP binds to pri-129-1 transcripts in myeloid cells.

(**a**) Immunoblot showing the IP efficiency of the KSRP antibody. **(b)** RIP-PCR analysis of the pri-129-1 transcript during PMA-induced monocytic differentiation. **(c)** qRT-PCR analysis of the expression of the pri-129-1 transcript in 293TN cells transfected with the pCMV6-129-MS2 plasmid, and the recruitment of the pri-129-1 transcript in MBP-MS2 IP. Three technical replicates from a single experiment. **(d)** qRT-PCR analysis of pri-129-1 expression after cells were transfected with different concentrations of the pCMV6-129-MS2 plasmid. Three technical replicates from a single experiment. **(e)** Immunoblot analysis of the precipitation of KSRP by 129-MS2 using a 23b-MS2 concentration gradient competitive RNA pull-down assay. **(f)** Immunoblot analysis of KSRP expression in the concentration-dependent 129-MS2 IP.

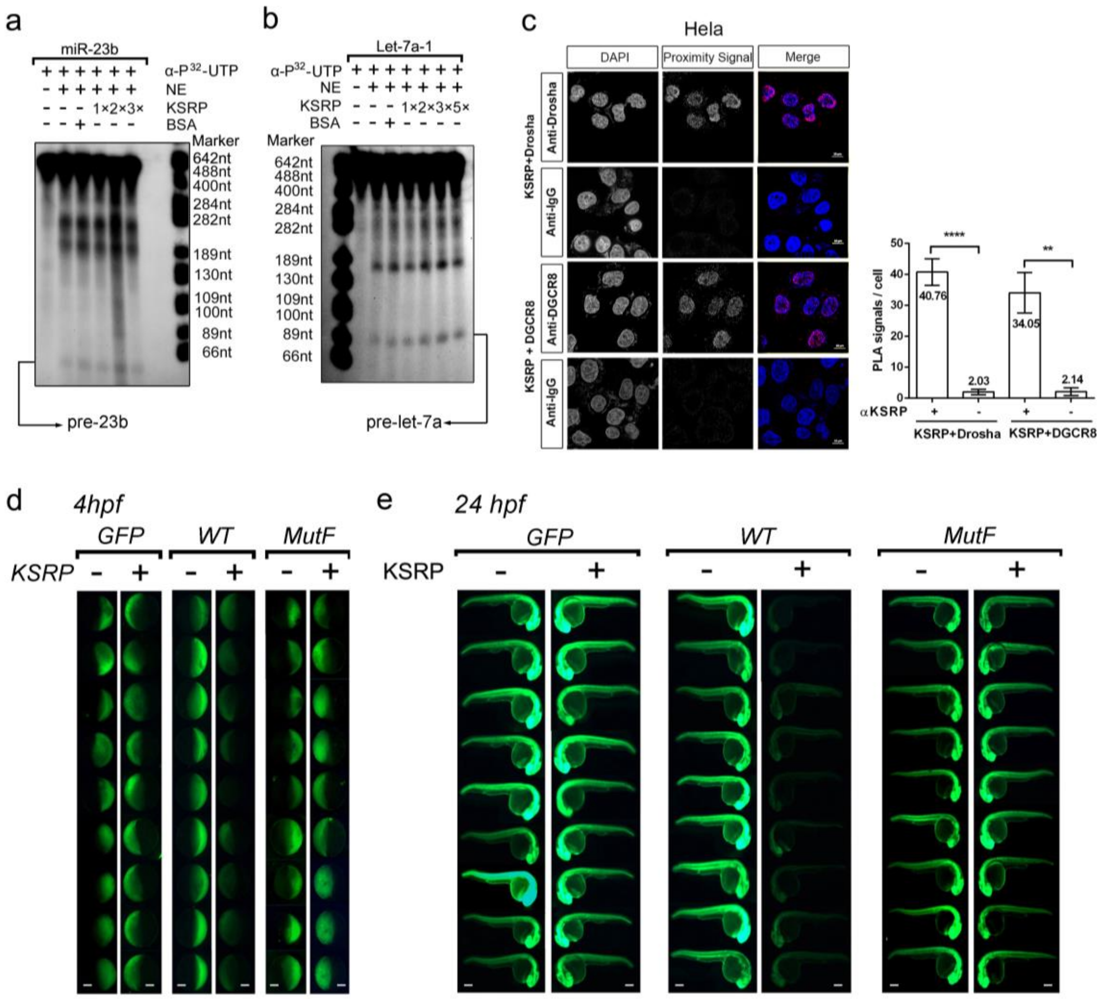

Supplementary Figure 5. KSRP promotes pri-129-1 processing.

(a) *In vitro* pri-miR-23b processing assay. **(b)** *In vitro* pri-let7a-1 processing assay. **(c)** HeLa cells were reacted with anti-KSRP and anti-Drosha or anti-DGCR8 for detection, or with anti-KSRP and anti-IgG as negative controls. PLA was performed to visualize KSRP-microprocessor interacting complexes: each red spot represents a single protein-protein interaction. Nuclei were stained with DAPI. Cells were analyzed by confocal immunofluorescence microscopy Zeiss LSM780 (Magnification, 630x). The number of fluorescent red dots was quantified on at least 100 cells (rigons ≥ 3) per condition, using the Blobfinder V3.2 software. Results are shown as mean of the number of PLA dots per cell. **(d, e)** KSRP promotes pri-129-1 processing in zebrafish; images were captured at 4 hpf (**d**) and 24 hpf (**e**). Scale bar = 200 µm. Data are shown as means ± s.d..***P*<0.01, ****P*<0.001, Student's *t*-test.

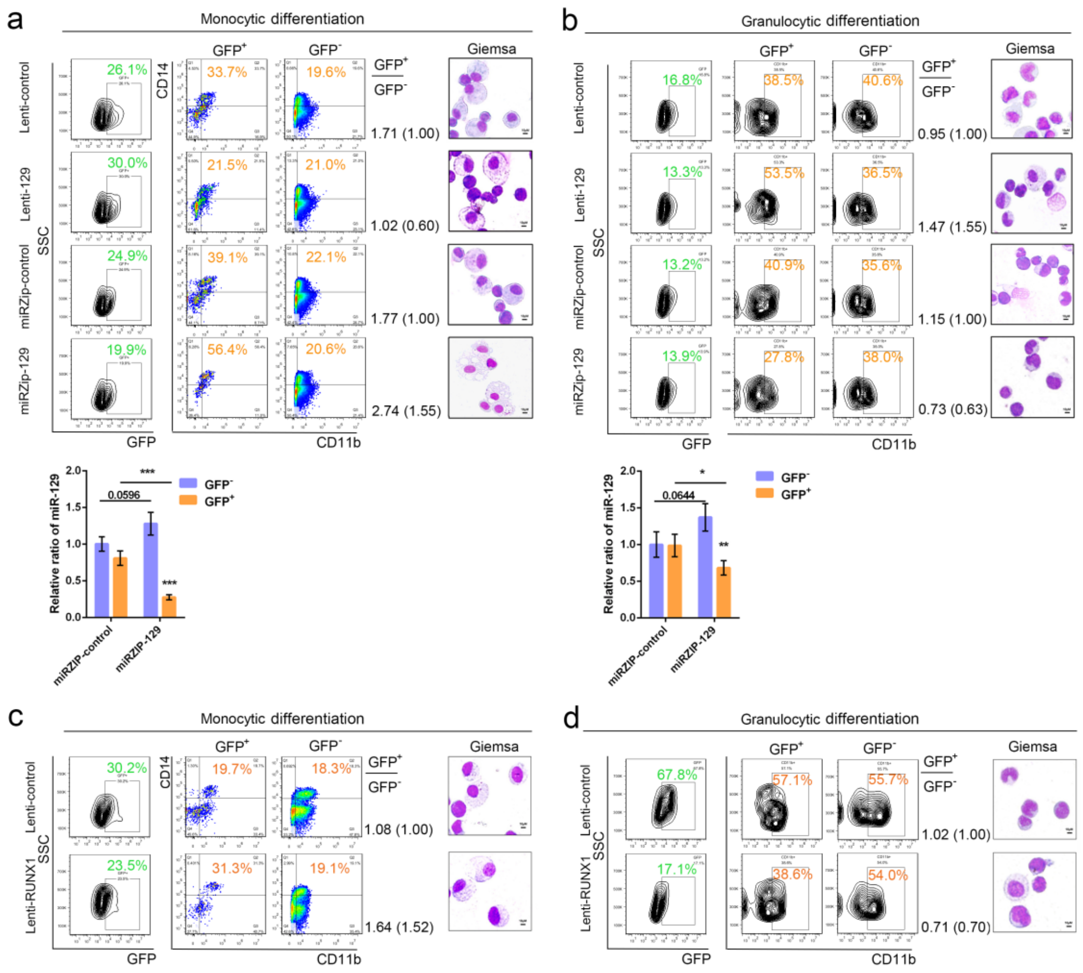

Supplementary Figure 6. miR-129 regulates monocytic and granulocytic differentiation by targeting RUNX1.

(a & b) CD34$^+$ HPCs were transduced with lenti-129 or lenti-control, or miRZIP-129 or miRZIP-control for 24 h, and then cultured for 15 days to allow cells to differentiate into monocytes (a) or granulocytes (b). FACS analysis of the CD14$^+$CD11b$^+$ population in monocytes and CD11b$^+$ population in granulocytes as well as the morphology (May-Grunwald Giemsa staining) of HPCs on day 15. Magnification, 40x. The bar represents 10 µm. Cell percentages in both GFP-positive and GFP-negative cells, as well as their ratio were shown. Relative miR-129 expression in GFP+ and GFP- population from miRZIP-129 or miRZIP-control transduced HPCs were analyzed by qPCR on day 15. **(c & d)** CD34$^+$ HPCs were transduced with lenti-RUNX1 or lenti-control for 24 h, and then cultured for 15 days to allow cells to differentiate into monocytes (c) or granulocytes (d). FACS analysis of the CD14$^+$CD11b$^+$ population in

monocytes and $CD11b^+$ population in granulocytes as well as the morphology (May-Grunwald Giemsa staining) of HPCs on day 15. Magnification, 40x. The bar represents 10 μm. Cell percentages in both GFP-positive and GFP-negative cells, as well as their ratio were shown. Three technical replicates from a single experiment. Data are shown as means ± s.d.. *P<0.05, ***P<0.001, Student's t-test.

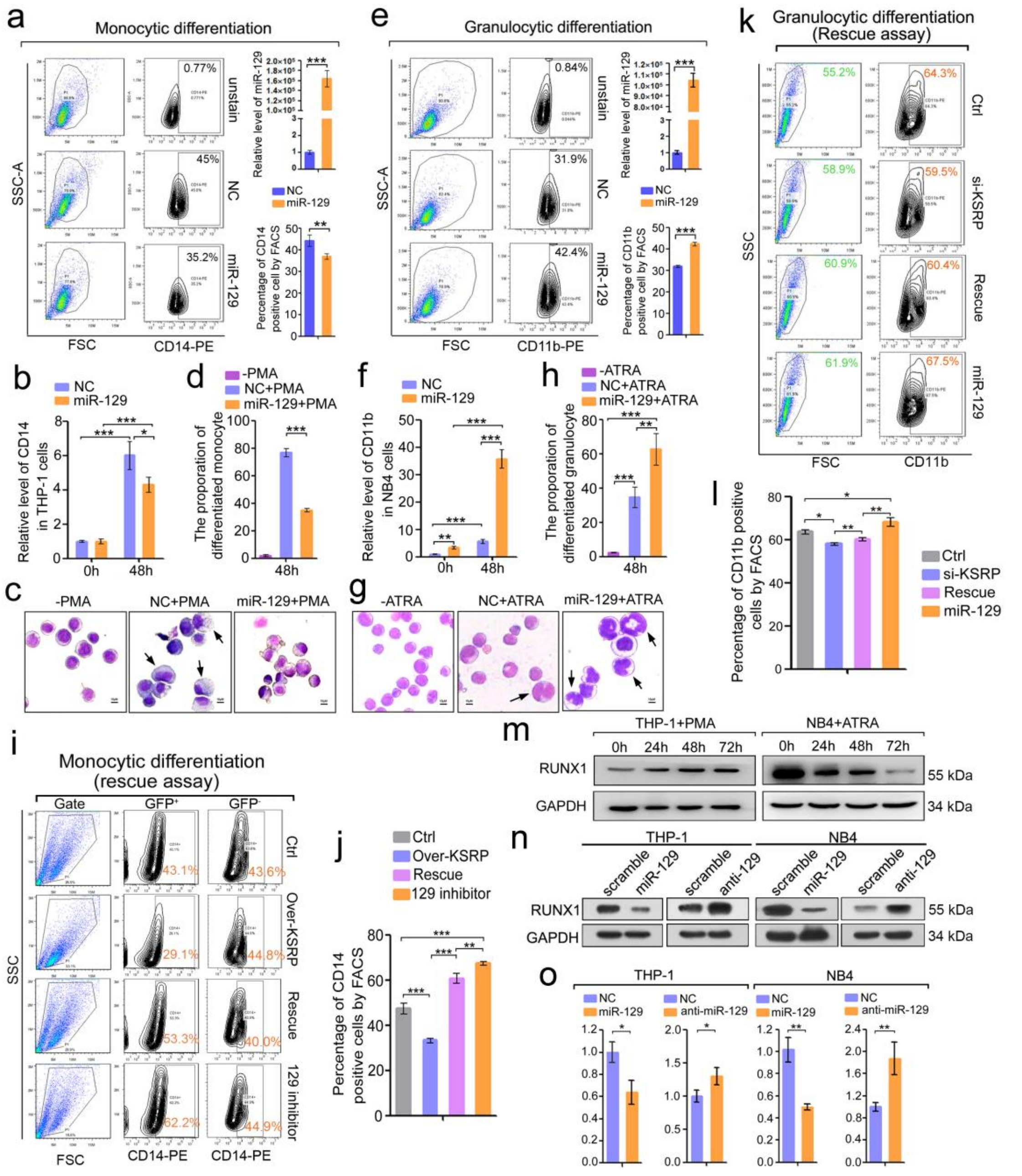

Supplementary Figure 7. miR-129 regulates monocytic and granulocytic differentiation in cell lines by targeting RUNX1.

(a-d) THP-1 cells were transfected with the miR-129 mimic or control mimic and induced to differentiate into monocytes with PMA for 48 hours. (**a**) FACS analysis of CD14 expression. (**b**) qRT-PCR analysis of *CD14* expression. (**c**) May-Grunwald Giemsa staining of cell morphology. The differentiated monocytes are indicated with arrows. Magnification, 40x. (**d**) Statistical analyses of the differentiated monocytes were performed by counting the cells shown in (c). Three technical replicates from a single

experiment. **(e-h)** NB4 cells were transfected with the miR-129 mimic or control mimic and induced to differentiate into granulocytes with ATRA for 48 hours. (**e**) FACS analysis of CD11b expression. (**f**) qRT-PCR analysis of *CD14* expression. (**g**) May-Grunwald Giemsa staining of cell morphology. The differentiated granulocytes are indicated with arrows. Magnification, 40x. (**h**) The statistical analysis of the differentiated granulocytes was performed by counting the cells shown in (g). Three technical replicates from a single experiment. **(i)** The rescue assay was performed by transfecting THP-1 cells with a combination of KSRP (or control) and miR-129 inhibitor (or control), followed by monocytic induction for 48 h. FACS analysis of $CD14^{+}CD11b^{+}$ cells in GFP-positive and GFP-negative population. **(j)** Statistical analysis of the three independent FACS experiments shown in (i). Three technical replicates from a single experiment. **(k)** The rescue assay was performed by transfecting NB4 cells with a combination of si-KSRP (or control) and miR-129 mimic (or mimic control), followed by granulocytic induction for 48 h. FACS analysis of CD11b expression. **(l)** Statistical analysis of the three independent FACS experiments shown in (k). Three technical replicates from a single experiment. **(m)** Immunoblot analysis of RUNX1 expression in THP-1 cells undergoing monocytic differentiation (left) or NB4 cells undergoing granulocytic differentiation (right). **(n)** The expression of RUNX1 in THP-1 and NB4 cells transfected with the miR-129 mimic (or mimic control) or miR-129 inhibitor (or inhibitor control), respectively, was analyzed by immunoblotting. **(o)** qRT-PCR analysis of the *RUNX1* mRNA levels in THP-1 and NB4 cells with modified miR-129 expression. Three technical replicates from a single experiment. Data are shown as means ± s.d.. $^{*}P<0.05$, $^{**}P<0.01$, $^{***}P<0.001$, Student's *t*-test.

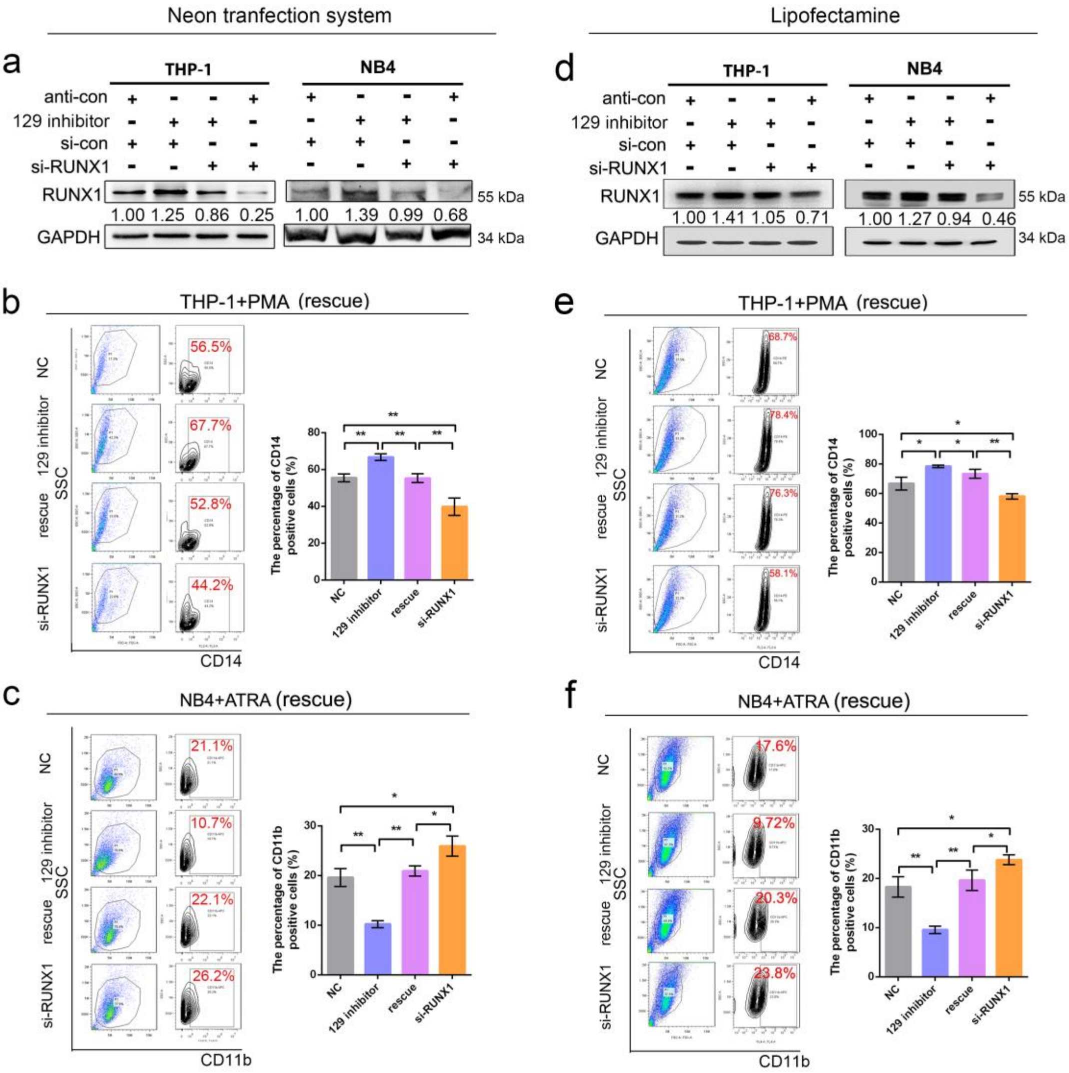

Supplementary Figure 8. miR-129 targets RUNX1 in myeloid cells.

The rescue assay was performed by transfecting THP-1 or NB4 cells with a combination of miR-129 inhibitor (or control) plus si-Runx1 (or control), followed by monocytic or granulocytic induction for 48 h. Cells were transfected with Neon transfection system (a-c) or lipofectamine (d-f). **(a, c)** Immunoblot analysis of RUNX1 expression. **(b, d)** FACS analysis of CD14 expression in THP-1 cells and statistical analysis of the three independent FACS experiments. Three technical replicates from a single experiment. **(c, f)** FACS analysis of CD11b expression in NB4 cells and statistical analysis of the three independent FACS experiments. Three technical replicates from a single experiment. Data are shown as means ± s.d.. *P<0.05, **P<0.01, Student's *t*-test.

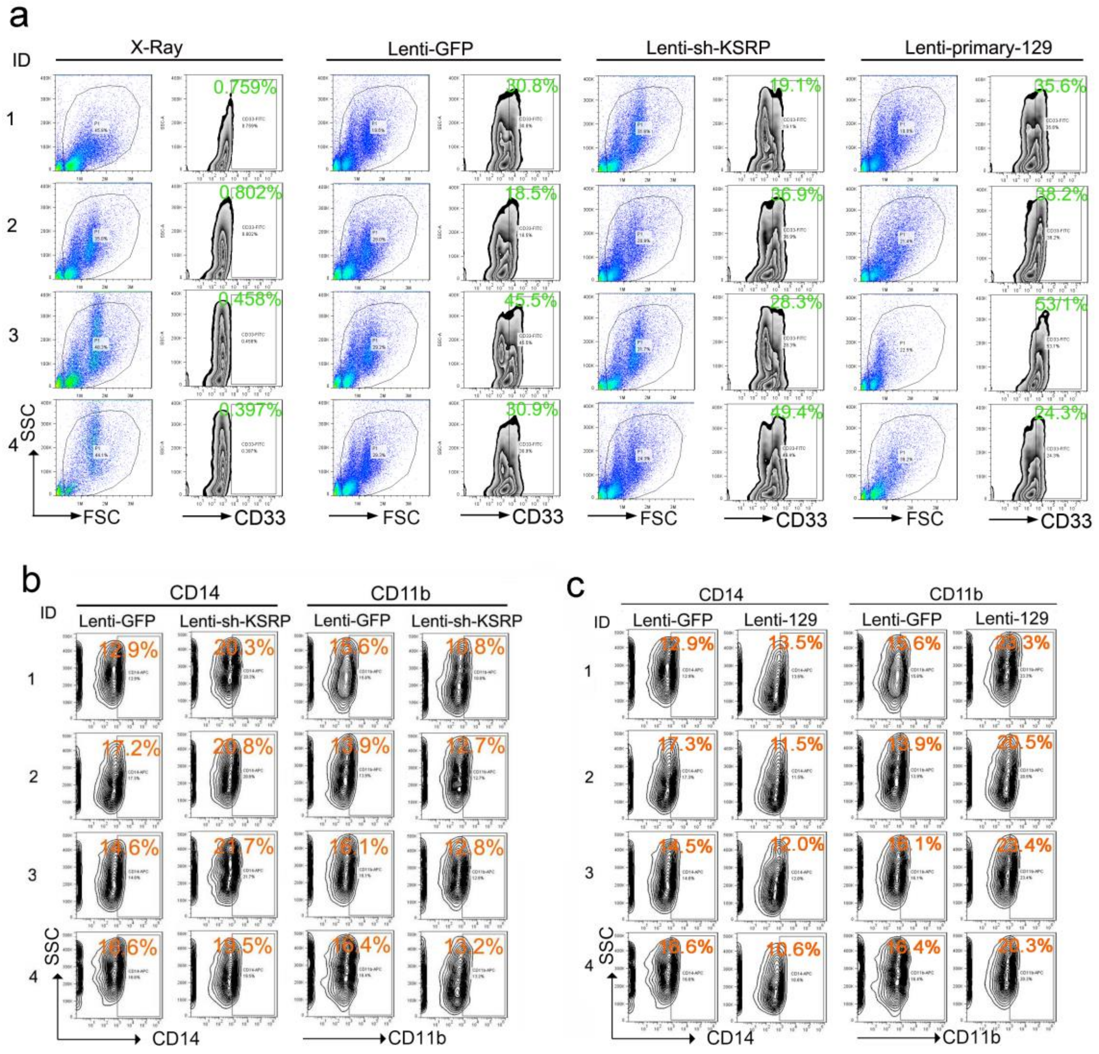

Supplementary Figure 9. Analysis of the transplanted mice.

(a) FACS analysis of the CD33^{+} populations in BM from transplanted or untransplanted (X-ray) mice. **(b)** The CD14^{+} and CD11b^{+} populations in BM from mice transplanted with lenti-sh-KSRP- or lenti-GFP-transduced HPCs were monitored by FACS. **(c)** The CD14^{+} and CD11b^{+} populations in BM from mice transplanted with lenti-129- or lenti-GFP-transduced HPCs were monitored by FACS.

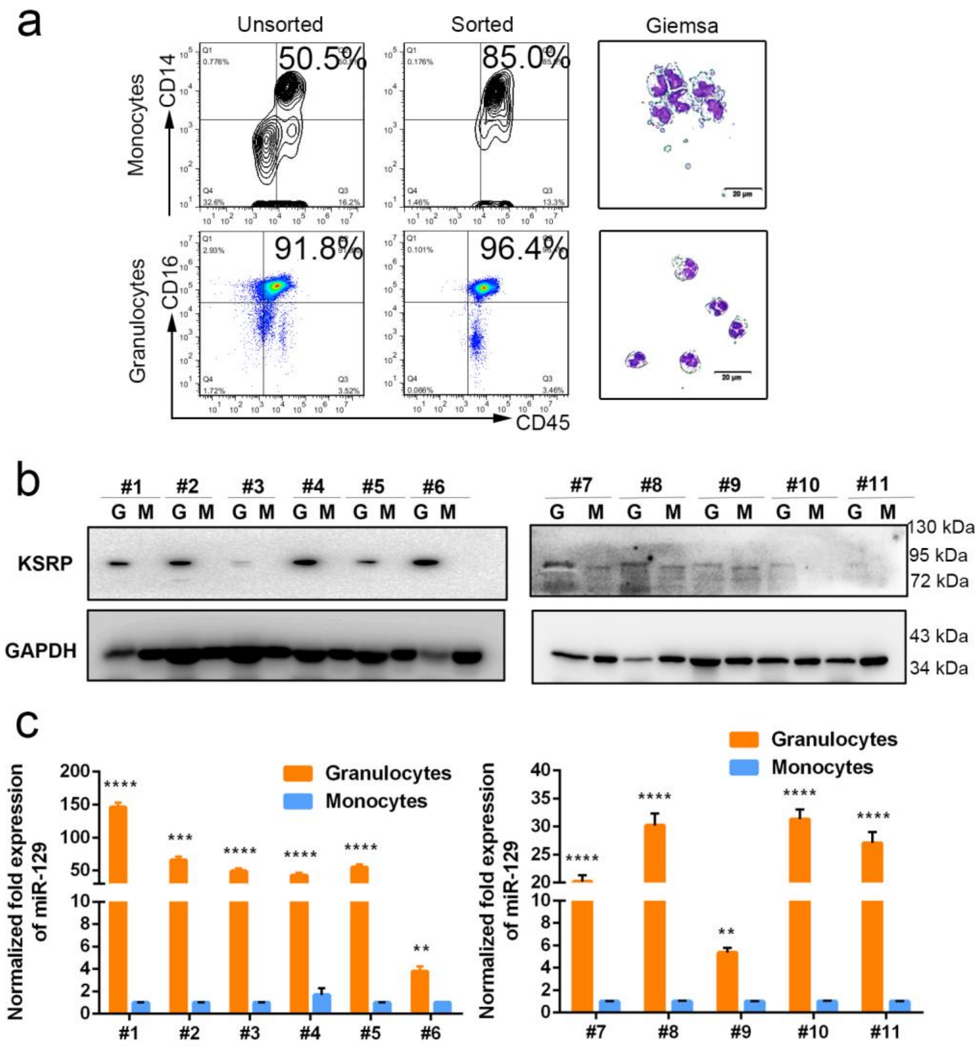

Supplementary Figure 10. KSRP protein and miR-129 levels in monocytes and granulocytes.

Monocytes and granulocytes were isolated from a total of 11 buffy coat samples derived from normal human peripheral blood. **(a)** Representative sorting efficiency of monocytes and granulocytes was analyzed by flow cytometry and Giemsa staining. **(b & c)** KSRP protein expression was detected by Western blot and miR-129 expression was analyzed by qPCR in the 11 samples. In each subject, the expression of miR-129 in granulocytes was normalized to that in monocytes. Data are shown as means ± s.d.. **P<0.01, ***P<0.001, ****P<0.0001, Student's t-test.

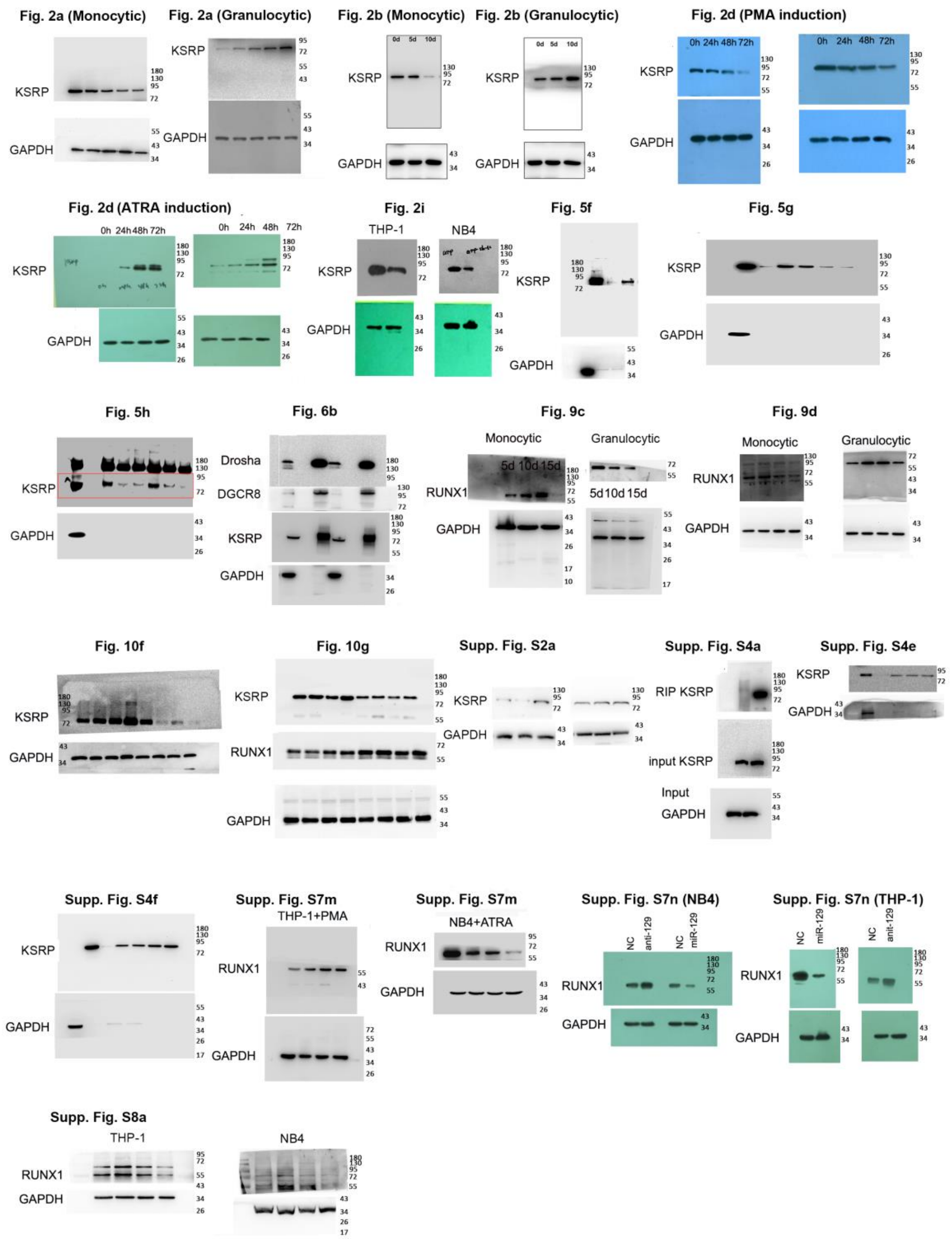

Supplementary Figure 11. Unprocessed Western Blots.

Unprocessed scans or pictures of Western blots are provided for main Figures as indicated.

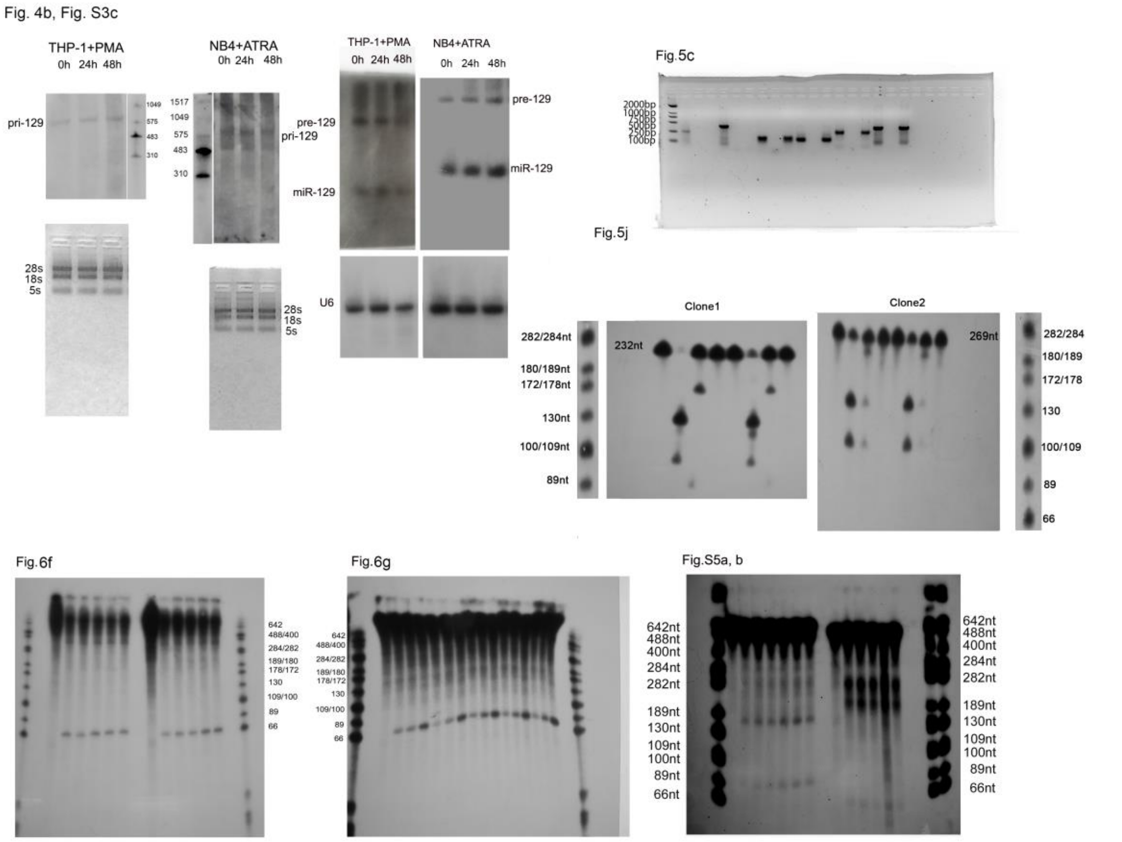

Supplementary Figure 12. Unprocessed Northern Blots and Agarose Gel Electrophoresis.

Unprocessed pictures of Northern blots and agarose gel electrophoresis of PCR products are provided for figures as indicated.
