# Supplementary material for: KSRP specifies monocytic and granulocytic differentiation through regulating miR-129 biogenesis and RUNX1 expression
Source: Nat Commun. 2017 Nov 10;8:1428. doi: 10.1038/s41467-017-01425-3 (PMC5681548; doi:10.1038/s41467-017-01425-3)
Supplement: Supplementary file 3 — Description of Additional Supplementary Files [file 41467_2017_1425_MOESM3_ESM.pdf]

**Description of Additional Supplementary Files**

**File Name: Supplementary Data 1**
Description: **DEGs in cells from batch 1 or batch 2 undergoing monocytic/granulocytic differentiation with confident assignments.** * The most likely expression path of a DEG in cells undergoing monocytic/granulocytic differentiation for 5, 10, and 15 days. ** The maximum posterior probability of a gene belonging to its corresponding expression path.

**File Name: Supplementary Data 2**
Description: **Expression (FPKM) of montonically upregulated or downregulated DEGs in cells from batch 1 or batch 2. RBPs are marked in red.** * In this column, 'Up' or 'Down' represents a gene that is monotonically upregulated or downregulated in the corresponding differentiating cells, whereas 'EE' represents a gene that is not a DEG in this culture.

**File Name: Supplementary Data 3**
Description: **Expression (FPKM) and clusters of 646 common DEGs in the intersections of batch 1 and batch 2.** * In this column, 'Up' or 'Down' represents a gene that is monotonically upregulated or downregulated in the corresponding differentiating cells, whereas 'EE' represents a gene that is not a DEG in this culture.

**File Name: Supplementary Data 4**
Description: **Expression (FPKM) of common RBPs in the intersections of batch 1 and batch 2.** * In this column, 'Up' or 'Down' represents a gene that is monotonically upregulated or downregulated in the corresponding differentiating cells, whereas 'EE' represents a gene that is not a DEG in this culture.

**File Name: Supplementary Data 5**
Description: **Expression of the miRNAs upon KSRP overexpression vs. the control.** Relative expression of miRNAs for which the expression of the mature miRNA was increased and the expression of the pri-miRNA decreased upon KSRP overexpression is listed.

**File Name: Supplementary Data 6**
Description: **Expression of the 16 candidate miRNAs upon KSRP overexpression vs. the control.** The 16 miRNAs were selected based on the following criteria: changes in mature miRNA expression > 1.3; changes in pri-miRNA expression < 0.8; relative expression < 1000.

**File Name: Supplementary Data 7**
Description: **Primers and siRNAs.** Primers used for constructing plasmids, RACE primers, mRNA qPCR primers, RIP-qPCR primers and siRNA sequences used in this study are listed in the worksheet tables.

**File Name: Supplementary Data 8**
Description: **R scripts.** R code was designed for classifying differential expressed genes from EBSeq-HMM result into different expression patterns.
